# Features of DNA Repair in the Early Stages of Mammalian Embryonic Development

**DOI:** 10.3390/genes11101138

**Published:** 2020-09-27

**Authors:** Evgenia V. Khokhlova, Zoia S. Fesenko, Julia V. Sopova, Elena I. Leonova

**Affiliations:** 1 Institute of Translational Biomedicine, St. Petersburg State University, 199034 St. Petersburg, Russia; evkhokhlova95@gmail.com (E.V.K.); zozoya07@mail.ru (Z.S.F.); sopova@hotmail.com (J.V.S.); 2 Institute of Cytology of the Russian Academy of Sciences, 194064 St. Petersburg, Russia; 3 Laboratory of Amyloid Biology, St. Petersburg State University, 199034 St. Petersburg, Russia; 4 Preclinical Research Center, University of Science and Technology, 1 Olympic Ave, 354340 Sochi, Russia

**Keywords:** DNA repair, BER (base excision repair), NER (nucleotide excision repair), MMR (mismatch repair), DSBR (double strand break repair), HR (homologous recombination), NHEJ (nonhomologous end joining), MHEJ (microhomologies end joining), oocyte, zygote, blastocyst

## Abstract

Cell repair machinery is responsible for protecting the genome from endogenous and exogenous effects that induce DNA damage. Mutations that occur in somatic cells lead to dysfunction in certain tissues or organs, while a violation of genomic integrity during the embryonic period often leads to death. A mammalian embryo’s ability to respond to damaged DNA and repair it, as well as its sensitivity to specific lesions, is still not well understood. In this review, we combine disparate data on repair processes in the early stages of preimplantation development in mammalian embryos.

## 1. Introduction

DNA repair during the early stages of embryonic development has one of the most significant effects on embryonic fate [1]. In the early embryonic stages of development, cells differ in their threshold of sensitivity to endogenous and exogenous factors [2]. However, to preserve and maintain the integrity of the genome, cells activate complex DNA repair mechanisms. It is believed that all major DNA repair pathways function in embryos. Repair proteins interact with cell cycle control proteins to stop the cell cycle if DNA is damaged, allowing the repair complexes time to fix the damaged DNA. If the damage is too substantial, and it is impossible to repair the DNA, a proapoptotic pathway is activated, resulting in cell death [3]. The mechanisms of regulation and functioning of repair systems are well studied in somatic cells. However, less is known about their activities during early embryonic development. Several reports have shown that early embryos and embryonic stem (ES) cells lack functional cell cycle control checkpoints, and DNA synthesis and cell division continue in the presence of damaged DNA. Ineffective activation of cell cycle checkpoints and suppression of apoptotic pathways in early embryos is associated with a shortened cell cycle, helping to ensure that the first embryonic cell division occurs, even under adverse conditions [4]. Thus, this review aims to analyze the literature and compare the role of repair systems at different stages of early mammalian embryonic development from the oocyte to the preimplantation blastocyst.

## 2. Oocyte Repair

**Figure 1:**
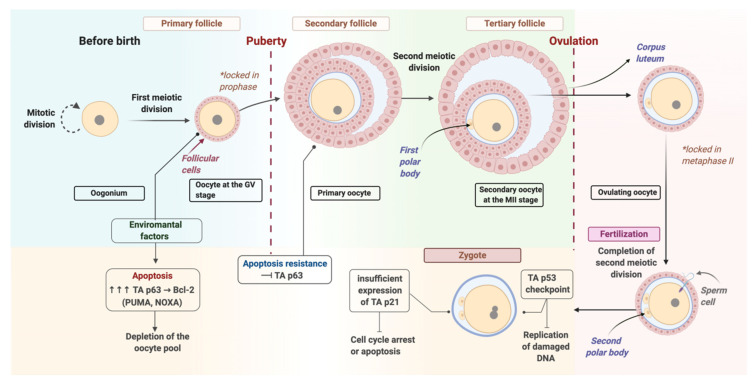
The process of differentiation in female germ cells. Created with BioRender.com.

Oocytes are one of the longest-living cells in the body, remaining at rest for many months (mouse) or decades (humans) [5]. During this time, they are exposed to exogenous and endogenous factors that cause damage to the DNA structure. DNA double strand breaks (DSBs) accumulate with age in primary follicle oocytes due to cellular metabolism and oxidative stress [6]. Factors such as γ-radiation, chemotherapy, and adverse environmental influences lead to the formation of DSBs in oocytes during the primary follicular stage [7,8,9]. In each of these cases, DSBs induce oocyte death if the damaged DNA is not repaired. This leads to depletion of the oocyte pool in the follicles, premature ovarian failure, infertility, and early menopause. Primary follicular oocytes at the germinal vesicle (GV) stage are more susceptible to DNA-damaging agents and are more prone to apoptosis compared to somatic cells and more mature MII stage oocytes [10]. This might be associated with the development of a highly sensitive apoptotic response since it is crucial to eliminate oocytes with damaged DNA to protect the germline [11]. Therefore, DNA damage control checkpoints are activated when the cell cycle stops during meiosis I, facilitating removal of oocytes with DNA that has not been restored after meiotic recombination. Members of the p53 family have been identified as critical regulators of apoptosis activity in oocytes during the GV stage [12]. According to published data, the *TAp63* gene is highly expressed in primary follicle oocytes and is an essential mediator of induced DNA damage response in oocytes due to transcriptional activation of proapoptotic members of the Bcl-2 family, PUMA and NOXA [13]. Interestingly, *TAp63* expression is suppressed when oocytes exit the follicle, which may partially explain why mature MII oocytes are more resistant to apoptosis due to DNA damage than oocytes in the GV stage (Figure 1) [14].

Furthermore, mature MII oocytes have a broader expression profile of mRNA encoding repair proteins compared to GV oocytes. Nevertheless, starting from the oocyte at the GV stage, expression of genes encoding proteins for all repair systems has been observed [15]. Extensive expression of repair genes corresponds to the oocyte’s ability to recognize and repair DNA damage from the earliest stages of development [16]. In 2009, Jaroudi et al. demonstrated that in humans, mRNA levels of most repair genes in oocytes are higher than in blastocysts, which is explained by the accumulation of a sufficient amount of mRNA to ensure preservation of the genome before and after fertilization until the zygotic genome is activated [17]. DNA repair transcripts that accumulate in human oocytes play an essential role in chromatin remodeling and maintenance of chromatin integrity during fertilization [18]. Transcripts of all DNA repair pathways, including base excision repair (BER), mismatch repair (MMR), nucleotide excision repair (NER), double strand break (DSBs) repair are presented in oocytes at the GV and MII stages in mouse, monkey, and human [17,19,20]. DNA glycosylase is involved in base excision repair in oocytes and zygotes and exhibits significantly higher levels in MII oocytes than in oocytes at earlier stages [21]. Similarly, expression levels of the *XPC* gene involved in nucleotide excision repair in MII oocytes is significantly higher than in GV oocytes [22]. Expression of the mismatch repair gene *MSH2* was also higher in oocytes of stage MII compared with oocytes in stages GV and MI [21]. *ATM* and *ATR* DNA repair markers are actively expressed during oocyte maturation, *ATR* expression is primarily manifested in immature oocytes during meiosis I. In addition, the DNA repair marker *BARD1* is highly expressed during oocyte maturation [23]. The BARD1-BRCA1 heterodimer is considered as an E3 ubiquitin ligase. Studies by Gasca et al. found that the E3 ubiquitin ligase multiprotein complex containing BARD1, BRCA1, and BRCA2 is involved in the maturation of human oocytes [24]. These proteins play a crucial role in regulating cell cycle development, DNA repair, and gene transcription [25]. Mutations in *BRCA* genes lead to an impaired ability to repair DNA DSBs and cause premature oocyte aging, apoptosis, and disturbances in meiotic divisions [26]. *BRCA1* and *BRCA2* homozygous deletion in mice results in embryonic lethality. Heterozygous deletion of *BRCA1* in mice results in impaired reproductive capacity, characterized by low follicle counts and an increase in the accumulation of DNA DSBs in surviving follicles relative to wild-type mice [10]. In experiments on rhesus monkeys, the RAD51 protein, which is involved in homologous DSB recombination, is expressed in oocytes. However, its expression decreases during oocyte maturation and then increases again at the eight-cell stage [21]. *RAD51* homozygous deletion in mice results in defective separation of sister chromatids, aneuploidy, and broken chromosomes at metaphase II [10].

According to Jaroudi et al., *RAD51* and *MSH2* are expressed at high levels in both oocytes and human blastocysts. The Ku70 is the DNA-binding component of the non-homologous end joining (NHEJ) repair machinery, it exhibits high expression levels in both oocytes and blastocysts [17]. Studying human oocytes using the single-cell sequencing method, the *DPYD* gene was discovered, which encodes the dihydropyrimidine dehydrogenase. Importantly, high NADP+ levels activated *DPYD* to enhance the repair of DNA double-strand breaks to maintain euploidy. In vivo high expression level of *DPYD* was observed in primary and secondary follicle oocytes, but this gene was not expressed in oocytes and preimplantation embryos. Expression of *DPYD* increased in primary and secondary follicles incubated in vitro and was dramatically upregulated in *in vitro* matured (IVM) oocytes. Furthermore, embryos from human IVM oocytes had more tiny chromosomal defects than those from human *in vivo* matured (IVO) oocytes. It has been shown that increasing the expression of *DPYD* may facilitate the repair process and overcome the risk of aneuploidy [27]. The compounds of *in vitro* culture medium may have an impact on embryo’s viability by dysregulation of some genes associated with DNA repair machinery [28]. Although all repair pathways function in oocytes, each pathway’s activation may vary depending on the stage of oocyte development [10]. Additionally, some reactions to DNA damage and DNA repair genes are overrepresented in the oocyte compared to the preimplantation embryo (from one-cell to blastocyst stages) [28]. This might reflect the particular importance of ensuring the integrity of the genome, especially after prolonged arrest during the first meiotic prophase. The repair pathways are redundant, and it is unknown whether all pathways are used simultaneously during oocyte development. Some mRNAs are translated in the oocyte, while others remain in a polyadenylated form until fertilization, maintaining a pool of repair proteins until the embryo genome is activated [29].

## 3. Repair at the Zygote Stage

The transformation of a fertilized oocyte into a zygote is an amazing process that occurs in the absence of transcription and depends on the mRNA accumulated in the oocyte during oogenesis. Sperm penetration causes activation of the egg, accompanied by the release of mRNA from complexes that block translation initiation [30]. In mouse oocytes, mRNAs contain elements of cytoplasmic polyadenylation (CPE) in their 3′-untranslated region [31]. Polyadenylated mRNA tails are linked to a repression translation complex containing CPE-binding protein (CPEB) and Maskin protein. Maskin binds eukaryotic translation initiation factor 4E (eIF4E), an interaction that excludes eIF4G and prevents formation of the eIF4F initiation complex [32]. During oocyte maturation, CPEB phosphorylation stimulates polyadenylation and recruitment of the poly (A)-binding protein bound to eIF4G, which helps to displace Maskin from eIF4E, thereby initiating translation [32]. The activation process contributes to the continuation of meiosis, the formation of pronuclei, and the translation of proteins necessary for the zygote [30]. Thus, the fusion of a terminally differentiated oocyte in metaphase II and the sperm cause complex changes, including chromatin remodeling and epigenetic reprogramming in the zygote. The most significant changes occur in the paternal genome, where compacted sperm chromatin is reorganized, protamines are replaced by histones, and various histones and DNA are demethylated in phases G1 and S [33]. At this stage in the chromosomes separately in the male and female pronuclei, active demethylation of the paternal DNA and passive demethylation of the maternal DNA occur [34]. Active demethylation of the paternal genome’s DNA includes mechanisms based on excision and DNA repair [35]. It should be noticed that cytosines in sperm DNA are highly methylated. Moreover, the majority of 5mC is demethylated, regardless of DNA replication, before the first cycle of zygotic cell division [36]. The mechanisms of active DNA demethylation in zygotes are not well understood. It has been suggested that different DNA-repair-based mechanisms are important for demethylation, e.g., processes initiated by DNA glycosylases, DNA methyl-transferases and DNA deaminases [37]. During zygotic reprogramming, 5mC is modified to 5-hydroxymethylcytosine (5hmC) using Tet3 hydroxylase. This oxidized cytosine is either removed or replaced with unmodified cytosine using BER or is passively removed during the next DNA replication cycle [38]. This leads to the breaking of DNA chains. Double- and single-stranded DNA breaks lead to the appearance of phosphorylated histone H2AX (γH2AX) and activation of other DNA repair systems [39]. Mature sperm cells are considered incapable of repairing DNA damage due to DNA compaction and decreased transcriptional activity. It has been suggested that the oocyte has the capacity to repair sperm DNA damage when the level of sperm DNA damage is less than 8%. Higher levels of sperm DNA damage are associated with a failure to reach the blastocysts phase and embryonic loss between the embryonic genome activation (EGA) and the blastocyst stages. [40]. There is evidence that human sperm has a basic BER pathway containing only OGG1 protein [41]. Being the first enzyme in the pathway, its presence is sufficient for sperm to detect and remove oxidized bases and residues of 8-OHdG, a standard oxidative stress product. Because the rest of the pathway is truncated, DNA obtained after removal of 8-OHdG must subsequently be restored by oocyte repair proteins after fertilization before the first cell division. However, it is interesting to note that expression of the *OGG1* gene in the oocyte at this stage is low [42]. Some authors consider the random complementarity of sperm cells and oocytes to be an elaborate mechanism for checking the compatibility of oocytes and fertilizing sperm since both must be involved in the restoration of oxidative damage to DNA [40]. It is believed that BER is the primary pathway for the restoration of oxidative damage in the zygotic genome. However, other repair systems can also contribute to the restoration of embryonic DNA at this stage of development [6]. DSB restoration in the zygote occurs using NHEJ and homologous recombination (HR) repair pathways. These pathways are not equally important during the cell cycle. The choice of DSB repair pathway depends on the developmental stage of the embryo and the cell cycle. NHEJ works throughout the cell cycle, while HR functions during the S/G2 stage. DSBs obtained by stopping replication are preferably restored using HR [43]. It is believed that at the zygotic stage, NHEJ plays an essential role in the restoration of sperm DSBs [44].

During fertilization male and female genomes fuse to form a zygote nucleus. Dynamic chromatin and protein rearrangements require post-translational modification, such as poly(ADP-ribosyl)ation, for the post-fertilization development. In addition, poly(ADP-ribosyl)ation of nuclear proteins is necessary for the detection of DNA strand breaks and the recruitment of repair factors to damaged sites. Poly(ADP-ribosyl)ation is catalyzed by enzymes of the poly(ADP-ribose) polymerase-1 (PARP) family. Cellular stress stimulates the activity of poly(ADP-ribose) polymerase-1 (PARP-1) on binding to DNA strand breaks, playing a key role in their repair. Inhibition of PARP-1 in oocytes impacts negatively on embryo survival [45]. Mice with homozygous mutations in the *PARP-1* gene are viable, although cells lacking *PARP-1* are hypersensitive to many DNA-damaging agents, and double homozygous knockout of *PARP-1* and *PARP-2* leads to embryonic mortality [45]. PARP-1 is one of the first proteins involved in the repair of DSBs based on micro-homologous end joining (MHEJ). PARP-1 has an affinity for the 3′-ends of DNA and competes with Ku proteins for binding to DNA upon damage, switching DSB repair pathways from NHEJ to MHEJ [46,47]. 

Normally, the paternal and maternal pronuclei in the mouse zygotes initiate DNA replication nearly synchronously between five and six hours after fertilization. But in response to DNA damage in the male pronucleus the zygote reacts by slowing down the replication of paternal DNA for up to 12 h, ultimately leading to a halt in embryonic development. Moreover, the replication delay in the female pronucleus does not occur. Pronuclei act independently, stopping DNA synthesis in response to DNA damage in only one pronucleus. The lack of synchronization in DNA replication between the two pronuclei in response to damage in the paternal DNA leads to two pronuclei at different stages of DNA replication being in the same cell [48]. Low expression of *CDKN1A* (p21) gene at the early embryo stage may result in inability of the embryo to respond to DNA damage by stopping the cell cycle. p21 is a target of p53 and mediates p53-dependent cell cycle arrest or apoptosis. The p53-dependent S-phase checkpoint functions at the zygotic stage to inhibit replication of damaged DNA [16]. p21-mediated cell cycle arrest occurs later, preventing delayed chromosome damage. Thus, during early development, embryos are protected by mechanisms regulated by p53 and p21 [49]. The first signs of apoptosis, such as cytoplasmic fragmentation, in the case of excessive unrepaired DNA damage, appear only at the two-cell stage in mice and four-cell stage in humans. However, other signs of apoptosis, including condensation of chromatin and cytoplasm with subsequent DNA degradation and nuclear fragmentation, are not observed until the morula and blastocyst stages (Figure 1) [48].

## 4. Repair at the Cleavage and Blastocyst Stages

**Figure 2:**
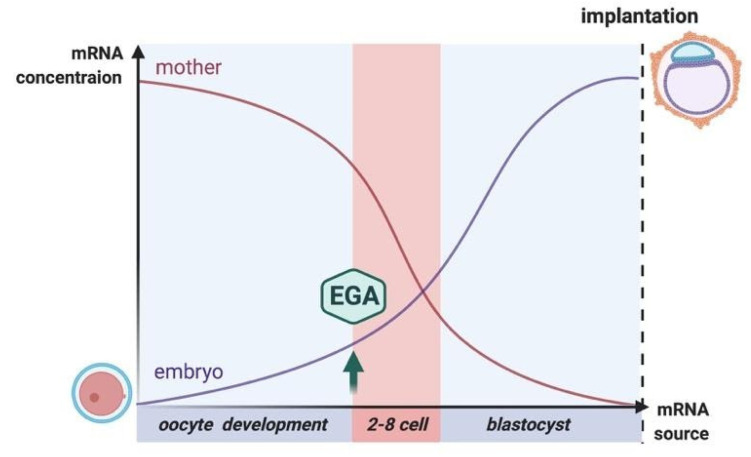
The source and dynamics of mRNA expression during early embryonic development. EGA—embryonic genome activation. Created with BioRender.com.

Human embryos at cleavage state show a high level of postzygotic chromosomal mosaicism, including aneuploidy and polyploidy. Mosaicism for the paternal alleles most probably resulted from mutations in genes that are involved in DNA mismatch repair (MMR), e.g., *PMS2* and *MSH2* [50,51]. The gene expression profiles of oocytes and one-cell embryos are highly similar, at the two-cell stage the gene expression dramatically changes. The second change in gene expression of embryonic genomes occurs at the four to eight cell stage, preceding cell compaction at the morula stage, which explains the separation between the two-cell and eight-cell stages. Eight-cell embryos and blastocysts slightly differ in gene expression (Figure 2) [28].

Blastocyst formation is the first stage during which the differentiation of cells into two types occurs, characterized by differences in gene expression between inner cell mass (ICM) and trophectoderm cells (TE) cells [28]. Subsequently, TE forms tissues of the placenta, while ICM cells give rise to embryonic tissues. Since ICM cells give rise to cells forming a new organism, maintaining the integrity of the genome in these cells is crucial [1]. Due to the high replication rate and the beginning of cell differentiation in the blastocyst, the expression profile of DNA repair genes differs from oocytes. It is believed that all DNA repair pathways are present in both the human oocyte and in the blastocyst. However, some DNA repair genes are expressed in the blastocyst at lower levels compared to the oocyte, such as *MBD4, NEIL1, OGG1* (BER); *RAD50, RAD54B, RBBP8* (HR); *MSH3* (MMR); *ERCC5, GTF2H2, LIG1, RPA1* (NER) [17]. This may be explained by the fact that the oocyte contains maternal mRNA transcripts to maintain genome integrity before EGA [17]. During development, embryos acquire the ability to respond to factors that cause DNA damage by activating and regulating DNA repair and apoptosis genes, similar to what occurs in somatic cells [16]. Mutations in some DNA repair genes lead to defects in early and later stages, including fetal mortality, infertility, or cancer susceptibility in postembryonic development. However, the fact that not all mutations in repair genes delay embryonic development in the early stages of development indicates that some mechanisms of DNA repair are either redundant or play less active roles during early embryogenesis and rather, may be involved during later developmental stages. Detailed information about the primary patterns of expression of DNA repair genes during different stages of development is needed to understand the extent to which preimplantation embryos can react to and repair DNA damage and to what extent preimplantation embryos are selectively sensitive to certain forms of DNA damage [16]. This information is difficult to obtain because the concentration of mRNA expressed in early embryos is quite low. However, many protocols have recently been developed, for instance, single-cell sequencing, that allow detection of small amounts of mRNA in each individual cell. Recently, many articles using this method have appeared [52,53,54]. Another way to investigate repair pathways in the early stages of development is to investigate blastocyst-derived embryonic stem (ES) cells.

## 5. Embryonic Stem Cells

ES cells are pluripotent cells isolated from the inner cell mass of a blastocyst. ES cells are sensitive to DNA damage and easily undergo apoptosis, removing damaged cells from the pluripotent pool [55]. Additional evidence suggests that DNA damage can cause premature differentiation in these cells. ES cells have a robust set of DNA repair mechanisms [20]. In particular, ES cells maintain significantly higher expression levels of proteins associated with HR than their expression levels in differentiated cells. Additionally, it is believed that levels of HR proteins decrease as ES cells differentiate [56]. ES cells have very short G1 phase and long period of S phase time can promote the use of HR rather than NHEJ since many of the proteins involved in HR also participate in DNA replication [57]. Due to the short G1 phase, sister chromatids are available for efficient recombination-mediated repair in ES cells [55]. ES cells derived from mouse blastocysts (mES) synthesize constantly high level of HR proteins throughout the cell cycle [56]. It can be suggested that the active HR repair pathway is required for a rapid response to DNA breaks in ES cells [56]. HR proteins are thought to be highly expressed but remain inactive until posttranslational modifications are triggered in response to damage. In experiments conducting treatment of mES cells with DNA break-inducing agents, Rad51, Rad54, Exo1, and γH2AX proteins were redistributed and concentrated in the nucleus as discrete foci. Therefore, in response to DNA damage, Rad51, Rad54, and Exo1 proteins are immediately localized at the sites of DNA strand break regions. It is believed that Rad51, Rad54, and Exo1 are constantly present in mES cells, providing fast and efficient HR-mediated DNA repair when needed [56]. The MMR also seems to play an essential role in ES cells, determining the fate of the cell with respect to whether the cell follows the DNA repair pathway or undergoes apoptosis. High endogenous levels of MSH2 protein in ES cells promote apoptosis, while low levels promote DNA repair, as occurs in differentiated cells [55]. Even low levels of MSH2 protein can reduce the number of spontaneous mutations in ES cells compared to MSH2 knockout ES cells, suggesting that MMR plays a fundamental role in regulating the level of mutagenesis in preimplantation embryonic cells [55]. NER machinery of ES cells cannot repair damage after high UV doses resulting in a rapid induction of apoptosis [58]. It is also known that the G1/S DNA damage checkpoint in ES cells can be activated upon DNA damage and prevent the cell from passing into the S phase [56]. However, there are conflicting data on this subject. Other studies indicate that rapidly dividing embryos and mouse ES cells have a nonfunctional G1/S checkpoint. Therefore, they cannot stop at G1 in the presence of DNA damage [59]. It is believed that high levels of the protein phosphatase CDC25A and the downstream CHK1 effector modulate the efficacy of the G1/S checkpoint in murine ES cells [60]. The ubiquitin hydrolase DUB3/USP17L2 supports high levels of CDC25A in these cells by removing polyubiquitin chains. The *DUB3* gene itself is a target for two pluripotency factors, ESRRβ and SOX2, and its expression is regulated during development, followed by rapid suppression after cell differentiation [61]. Therefore, the nonfunctioning control point G1/S is characteristic of mouse ES cells that are inappropriately associated with the state of pluripotency [62]. However, in human ES cells, the G1/S checkpoint is active, while the S-phase checkpoint is inactive. This difference may be due to differences in mouse and human embryo cell cycle and differences in pluripotency maintenance. It may also be the reason for differences in the activation of the embryonic genome. In mouse, EGA occurs at the two-cell stage, while in human EGA occurs at the eight-cell stage. Following this hypothesis, it was recently reported that primates have less reliable mechanisms for genome surveillance than rodents [59].

## 6. Conclusions

In this review, we briefly survey the repair processes from the oocyte stage to the preimplantation stage of the blastocyst. In each of these stages, we see changes in gene expression patterns and the involved repair systems. Oocytes in the GV stage are more susceptible to DNA damage and more prone to apoptosis, while more mature stage II oocytes do not activate apoptosis and are more resistant to DNA damage [10]. At MII stage, oocytes actively accumulate transcripts of repair proteins to protect the genome from DNA damage during fertilization and the first division. After fertilization in zygotes, maternal mRNAs that accumulated in the oocyte are actively used to repair the sperm genome and to maintain DNA integrity in subsequent development. Antiapoptotic proteins that protect the early embryo from death are also active at this stage because apoptosis at this stage would be fatal. However, in cases of critical levels of damage, developmental arrest occurs, accompanied by cell cycle arrest [48]. As the number of embryonic cells increases, the embryonic genome is activated, and maternal mRNAs are destroyed [18]. During this period, the embryo becomes more sensitive to external influences. However, when passing from the stage of a two-cell embryo to the blastocyst stage, its own repair proteins accumulate, and apoptotic systems that remove blastomeres with damage are activated [63].

As cells differentiate and lose their pluripotency, they acquire repair properties similar to somatic cells. An active change in the patterns of expression of repair genes during embryonic development indicates that repair processes are essential for normal functions at all stages of development. Repair processes in embryonic development are especially important. Currently, there are many gaps regarding the precise roles and timing of expression of some DNA repair genes in the early stages of embryonic development. The observed stage-specific variations in transcripts and expressed proteins of DNA repair genes indicate the difficulty in regulating these pathways during development.
